# PIEZO1 is downregulated in glenohumeral chondrocytes in early cuff tear arthropathy following a massive rotator cuff tear in a mouse model

**DOI:** 10.3389/fbioe.2023.1244975

**Published:** 2023-09-05

**Authors:** Devon E. Anderson, Katherine G. Broun, Paromita Kundu, Xingyu Jing, Xiang Tang, Christopher Lu, Alexander Kotelsky, Sandeep Mannava, Whasil Lee

**Affiliations:** ^1^ Center for Musculoskeletal Research, University of Rochester, Rochester, NY, United States; ^2^ Department of Orthopaedics and Physical Performance, University of Rochester, Rochester, NY, United States; ^3^ Department of Biomedical Engineering, University of Rochester, Rochester, NY, United States; ^4^ Department of Physiology and Pharmacology, University of Rochester, Rochester, NY, United States

**Keywords:** articular cartilage, chondrocyte, rotator cuff tear (RCT), cuff tear arthropathy (CTA), PIEZO1 channel

## Abstract

**Introduction:** A massive rotator cuff tear (RCT) leads to glenohumeral joint destabilization and characteristic degenerative changes, termed cuff tear arthropathy (CTA). Understanding the response of articular cartilage to a massive RCT will elucidate opportunities to promote homeostasis following restoration of joint biomechanics with rotator cuff repair. Mechanically activated calcium-permeating channels, in part, modulate the response of distal femoral chondrocytes in the knee against injurious loading and inflammation. The objective of this study was to investigate PIEZO1-mediated mechanotransduction of glenohumeral articular chondrocytes in the altered biomechanical environment following RCT to ultimately identify potential therapeutic targets to attenuate cartilage degeneration after rotator cuff repair.

**Methods:** First, we quantified mechanical susceptibility of chondrocytes in mouse humeral head cartilage *ex vivo* with treatments of specific chemical agonists targeting PIEZO1 and TRPV4 channels. Second, using a massive RCT mouse model, chondrocytes were assessed for mechano-vulnerability, PIEZO1 expression, and calcium signaling activity 14-week post-injury, an early stage of CTA.

**Results:** In native humeral head chondrocytes, chemical activation of PIEZO1 (Yoda1) significantly increased chondrocyte mechanical susceptibility against impact loads, while TRPV4 activation (GSK101) significantly decreased impact-induced chondrocyte death. A massive RCT caused morphologic and histologic changes to the glenohumeral joint with decreased sphericity and characteristic bone bruising of the posterior superior quadrant of the humeral head. At early CTA, chondrocytes in RCT limbs exhibit a significantly decreased functional expression of PIEZO1 compared with uninjured or sham controls.

**Discussion:** In contrast to the hypothesis, PIEZO1 expression and activity is not increased, but rather downregulated, after massive RCT at the early stage of cuff tear arthropathy. These results may be secondary to the decreased axial loading after glenohumeral joint decoupling in RCT limbs.

## Introduction

The rotator cuff is a group of four tendons from corresponding scapular muscles that coalesce onto the humerus around the shallow ball-and-socket glenohumeral joint to provide a wide range of upper extremity motion as well as dynamic joint stabilization through force coupling. Symptomatic rotator cuff disease is common and arises from either acute traumatic injury or chronic degeneration. The incidence of rotator cuff disease increases with age due to chronic tendon degeneration, leading to partial- or full-thickness tears (Lawrence et al., 2019). Over time, full-thickness tears are at risk of enlarging to a massive rotator cuff tear (>5 cm or involving 2 or more tendons), and the cuff no longer provides dynamic stabilization of the glenohumeral joint (Keener et al., 2015; 2019). The altered biomechanics lead to a distinct degenerative phenotype—cuff tear arthropathy (CTA)—characterized by eccentric articular cartilage wear and bony remodeling of the glenohumeral joint (Aumiller and Kleuser, 2015; Rugg et al., 2018). Prior work has shown distinct spatial differences in articular cartilage morphology of the humeral head in human specimens retrieved from arthroplasty and in the glenohumeral joint from a mouse model of CTA. In humans, the superior aspect of the humeral head exhibits tearing, fibrillation, and thinning of articular cartilage; whereas, articular cartilage at the center of the humeral head exhibits overall thickening, a multi-layered tidemark zone, and clustered chondrocytes (Toma et al., 2018). In the mouse, articular cartilage is also thickened in the central humeral head at early time points following a massive rotator cuff tear, which is hypothesized to correspond to a period of inflammation and remodeling in the altered mechanical environment (Zingman et al., 2017).

Articular cartilage of the glenohumeral joint resides in a complex and dynamic mechanical environment. Chondrocytes are intrinsically mechanosensitive across a wide range of mechanical loading due to the expression of mechanically activated (MA) ion channels (Xu et al., 2020; Delco and Bonassar, 2021; Gao et al., 2022). PIEZO1 is a calcium-permeating channel activated by mechanical stretch at the cell membrane (Coste et al., 2010; Coste et al., 2012). We have previously shown the critical role of PIEZO1 channels in calcium-mediated responses of chondrocytes against injurious loading and inflammatory cues *in vitro* using porcine and human knee articular cartilage (Lee et al., 2014; Lee et al., 2020; Lee et al., 2021). After Piezo1-specific knock-out with siRNA or PIEZO1-inhibiting peptide treatment (GsMTx4), chondrocytes significantly reduced both the high-strain induced Ca^2+^ influx *in vitro* and resultant injurious loading-induced cell death *in situ* (Lee et al., 2014). While PIEZO1 channels in chondrocytes are activated by hyper-physiologic injurious stimuli, Transient Receptor Potential Vanilloid 4 (TRPV4) channels are activated by physiological loading and mediate anabolic responses of chondrocytes (O’Conor et al., 2014; Servin-Vences et al., 2017; Lv et al., 2018; Chery et al., 2020; Nims et al., 2021; Gao et al., 2022; Savadipour et al., 2022). Mechanical wear and physical impingement of articular cartilage likely account for part of the mechanism to drive progressive degenerative changes associated with CTA; however, chondrocyte mechanotransduction and metabolism remains undefined in the setting of altered joint biomechanics following a full-thickness rotator cuff tear. A better understanding of the joint environment may provide opportunity for intervention to improve cartilage homeostasis and joint longevity following rotator cuff surgical repair, reconstruction, or augmentation.

The objective of this study was to elucidate chondrocyte mechanotransduction in the altered biomechanical environment following a massive rotator cuff tear to ultimately identify potential therapeutic targets to attenuate cartilage degeneration after rotator cuff repair. We hypothesized that a massive rotator cuff tear in a mouse model increases PIEZO1 expression and activity, making chondrocytes susceptible to mechanical injury.

## Methods

### *In situ* humeral head chondrocyte mechano-vulnerability

**FIGURE 1:**
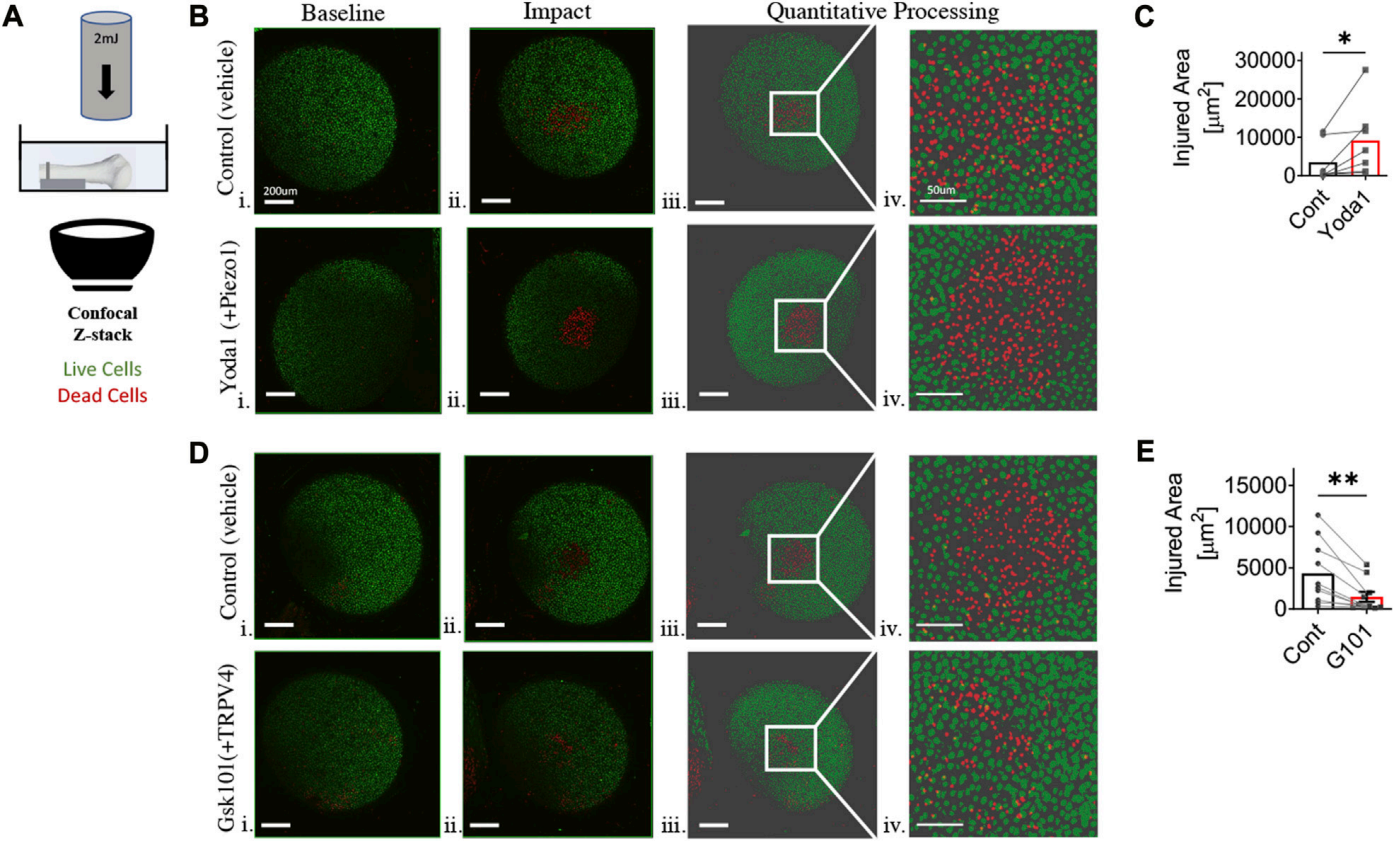
Chondrocyte mechano-vulnerability in humeral heads of C57BL6/J male mice. **(A)** Schematic of mechano-vulnerability set up. Humeral heads were imaged via fluorescence confocal microscopy before and after the 2mJ impact. The live and dead cells are indicated in red and green as shown. Cells outside of the sphericity of the humeral head are within the stump of the rotator cuff tendons and joint capsule, which was reflected during dissection. **(B)** Representative images of live/dead assay after 2mJ impact of humeral cartilage of contralateral control group (DMSO 0.02%) and with Yoda1, PIEZO1 specific agonist (10 μM) i. Micrograph of before impact, scale bar = 200 μm ii. Micrograph of after impact, scale bar = 200 μm iii. Quantitative processed image from MATLAB analysis of after impact, scale bar = 200 μm iv. Quantitative processed image from MATLAB analysis with increased magnification of injured region, scale bar = 50 μm. **(C)** Quantification of the area of cell death induced by 2mJ impact. Significantly increased injured area in Yoda1-treated group. *p < 0.05 with paired Student’s t-test. **(D)** Representative images of live/dead assay after 2 mJ impact of humeral cartilage of contralateral control group (DMSO 0.02%) and with GSK101, TRPV4 specific agonist (5 nM) i. Micrograph of before impact, scale bar = 200 μm ii. Micrograph of after impact, scale bar = 200 μm iii. Quantitative processed image from MATLAB analysis of after impact, scale bar = 200 μm iv. Quantitative processed image from MATLAB analysis with increased magnification of injured region, scale bar = 50 μm. **(E)** Quantification of area of cell death by 2 mJ impact. Significantly reduced injured area in GSK101-treated group. **p < 0.01 with paired Student’s t-test, *n* = 7-10.

Ethical approval was obtained by the institutional UCAR committee (Protocol #2019-008). Humeri were harvested from wild-type 12-22 week old C57Bl/6 male mice. Each specimen was placed in calcium imaging buffer with 2 mM calcium chloride and subsequently incubated with 10uM calcein-AM for 30 min at 37°C to indicate intracellular esterase activity of living cells. Specimens were then incubated with 10uM yoda1, a PIEZO1-specific agonist, 5 nM GSK101, a TRPV4-agonist, or control (DMSO 0.25%, *n* = 7-10/group) in calcium imaging buffer for 15 min. Specimens were then incubated with 1.5 nM ethidium homodimer-1 for 3 min to indicate loss of plasma membrane integrity in dead cells. The specimen was then arranged in a custom 3-D printed holding device such that the chondrocytes within the apex of sphericity of the humeral head were facing the glass well of a dish to be imaged on an inverted confocal microscope. A baseline confocal image with overlay of 100um Z-stacks was obtained with 488 and 594 nm wavelengths to image live and dead cells, respectively. The humerus was then transferred to a custom impact loading apparatus (Figure 1A). A 2mJ load was applied to the lateral aspect of the proximal humerus, such that the convex apex of the humeral head cartilage was impacted against the glass microscope dish. A 2mJ load was selected based on preliminary work by Kotelsky et al. (2019) to define mechano-vulnerability of distal femur and proximal humerus articular chondrocytes in the custom loading apparatus. The samples were again incubated in ethidium homodimer-1. A post-injury confocal image with overlay of 100 um Z-stacks was obtained. A custom MATLAB image processing algorithm was developed to quantify cell viability. The confocal images were processed as maximum intensity projections with threshold via Otsu’s method. Then, watershed segmentation, distance transform, and centroid approximation algorithms were created and applied to segment and quantify individual cells based on intensity values. This facilitated the cell counting process, allowed quantification of the total cell area, and allowed adjustment of the region of interest between before- and after-impact micrographs.

### Surgical model of a massive RCT

**FIGURE 2:**
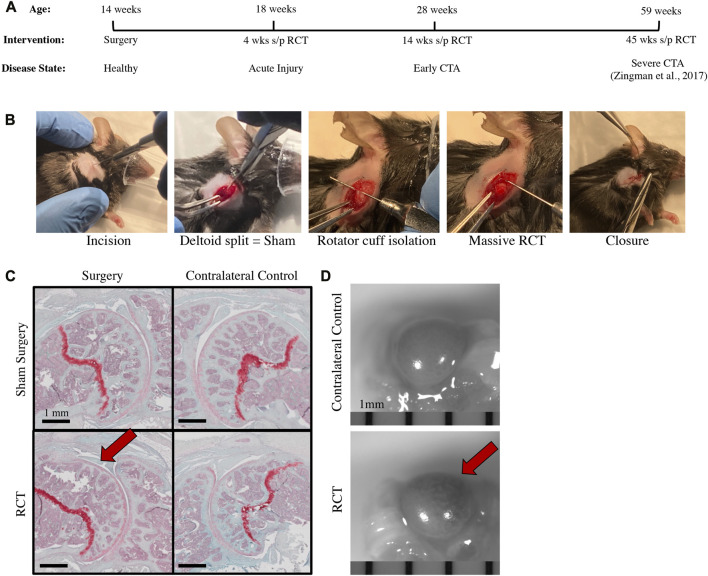
Rotator cuff tear (RCT)-induced mouse model of CTA with histologic & pathologic specimens 14 weeks post-injury. **(A)** Timeline of intervention, experimental analysis, and anticipated disease status with regards to OA including analysis of acute injury at 4 weeks post-injury, early CTA at 14 weeks post-injury, and reference to late-stage CTA at 45 weeks post-injury (Zingman et al., 2017). **(B)** Images representing surgical steps to produce massive RCT in mice. **(C)** Safranin-O histology of (*top left*) Sham, (*top right*) Sham Control, contralateral limb, (*bottom left*) RCT with humeral head flattening indicated by red arrow, and (*bottom right*) RCT Control, contralateral limb, scale bar = 1 mm. **(D)** Images of gross pathology specimens of (*top*) RCT Control, contralateral limb and (*bottom*) RCT with bone bruise indicated by red arrow, scale bar = 1 mm.

A massive rotator cuff tear (RCT) model was performed by surgical ligation and excision of the supraspinatus and infraspinatus tendons of the right shoulder of 14-week-old C57Bl/6 male mice through a deltoid splitting approach. Animals were sacrificed 4- or 14-weeks post-operatively (Figure 2A), which represent timing of an acute injury and early signs of CTA in a previously published mouse model, respectively (Zingman et al., 2017). In the surgical procedure (Figure 2B), mice were anesthetized with 2% isoflurane gas mixed with oxygen. A single dose of buprenorphine SR (0.5 mg/kg) was injected subcutaneously at the nape. The right shoulder was prepped using a chemical hair removal agent, followed by betadine solution for 2 min and a rinse with 70% ethanol. The remaining steps were performed under sterile technique. A #11 scalpel blade was used to sharply incise the skin and subcutaneous tissues in a longitudinal incision over the palpable border of the lateral shoulder. The deltoid fascia and muscle were sharply incised in line with the skin incision with care not to dissect distally along the humerus to protect the axillary nerve. A pair of forceps were then used to grasp the proximal humerus and pull laterally to place the glenohumeral joint on gentle tension. A 25G needle was passed under the supraspinatus and infraspinatus tendons. Spring scissors were used to sharply divide the tendons as proximally as possible, and the tendon stump was sharply removed with spring scissors. The deltoid fascia was then reapproximated with a single 8-0 vicryl suture in a simple stitch. The skin was closed with 5-0 nylon suture in simple stitches. The mice were recovered on a 37°C warmer prior to being returned to their home cage. Sham surgery was performed on control animals such that a deltoid splitting approach was used to dissect down to, but not ligate, the rotator cuff tendons. The contralateral (left) shoulder served as an uninjured control. Animals were monitored daily for 3 days post-operatively, and no adverse event were noted. They were then maintained in a standard unrestricted cage environment for the follow-up duration.

### Glenohumeral joint analysis status post RCT

Humeri were harvested and chondrocyte mechano-vulnerability was quantified before and after 2mJ impact according to methods above. Forequarter amputation of the fore limbs at the scapula was performed on each mouse bilaterally to keep the glenohumeral joint and associated muscular anatomy intact. Specimens were fixed in 10% neutral buffered formalin for 72 h prior to decalcification in 14% EDTA “Webb Jee” solution for 7 days. Specimens were then paraffin embedded and sectioned for resultant coronal orientation, with 7 um sections taken from the center of the glenohumeral joint. Sections were then deparaffinized and rehydrated through a series of xylene and alcohol baths. The central section from each joint was then stained with Safranin O to localize cartilaginous tissue, and humeral head cartilage was assessed for surgical and control joints.

### Quantification of PIEZO1 expression and activity

For PIEZO1 immunohistochemistry, deparaffinized and rehydrated slides were incubated in citrate buffer (10 mM sodium citrate, 0.05% Tween-20, pH 6.0) for 10 min at 95°C. Sections were blocked with 10% Normal Goat Serum (NGS) for 1 hour and incubated with anti-PIEZO1 antibody (1:200, rabbit polyclonal, Proteintech #15939-1-AP) overnight at 4°C. Following 30 min of wash with PBS containing 0.1% Triton-X (PBS-T) sections were incubated with goat anti-rabbit secondary antibody conjugated to Alexa Fluor 647 (1:1000, ThermoFisher Scientific) for 1 hour at room temperature. Slides were washed with PBS-T for 30 min and nuclei were stained with 4’,6-diamidino-2-phenylindole (DAPI; Sigma). The slides were mounted with Fluoromount-G™ (Invitrogen #00-4958-02). The images were captured using Olympus VS120 Virtual Slide Microscope and Visiopharm Image Analysis System. Chondrocyte PIEZO1 protein expression was quantified by measuring Integrated Density of each individual chondrocyte in ImageJ. Cells with circular cell shape were defined as region of interest (ROI). 20 cells at both humeral and glenoid were selected for each sample respectively after setting the threshold. The Integrated Density was then averaged for each side and summed up to represent the entire glenohumeral joint. The intensity was normalized to control.

### Calcium imaging

Cartilage from RCT limbs and contralateral control limb (left) were harvested from glenohumeral joint 14 weeks post-injury and were analyzed via ratiometric Ca^2+^ imaging (*n* = 4 mice). Cartilage samples were loaded with 0.0007 w/v% Fura-2 (Invitrogen) and 0.0625 w/v% F127 (PluronicTM) in 2 mM Ca^2+^ buffer solution for 40 min, then samples were buffer-washed for 10 min. Chondrocytes in superficial zones were imaged at 340nm/380 nm using ratiometric Ca^2+^ imaging system (Intracellular Imaging, Inc). Basal cytosolic Ca^2+^ levels ([Ca^2+^]_o_) were obtained by averaging before treatment. At 1 min, samples were treated with 40 μM Yoda1 (Tocris Bioscience, Inc.), a selective activator of the mouse and human mechanosensitive channel PIEZO1. Samples were imaged for 6 minutes. Delta cytosolic Ca^2+^ levels (Δ[Ca^2+^]_Yoda1_) were obtained by averaging the differences between [Ca^2+^]_o_ and maximum Ca^2+^ levels reached in responsive cells. A responsive cell was defined if it had a maximum calcium peak with a magnitude of at least three times higher than its 1-min pre-treatment Ca^2+^ level fluctuations in standard deviation.

### Quantification of PIEZO1 expression after non-invasive ACL injury in a mouse model

Wild-type 8-week-old C57Bl/6 male mice were subjected to bilateral ACL injury as previously described (Kotelsky et al., 2022). Four weeks after ACL injury, distal femoral cartilage was assessed for PIEZO1 expression with the immunohistochemistry methods described above, without variation, for comparison with uninjured control animals.

### Statistical analyses

Paired student’s t-tests were used to evaluate for differences between treatment and contralateral control groups, unpaired student’s t-tests were used to compare sham with surgical intervention, and ANOVA was used to compare multiple groups, with statistical significance set to *p* < 0.05. A minimum of three biologic replicates were used for each experiment.

## Results

### PIEZO1 activation increases chondrocyte mechano-vulnerability and TRPV4 activation decreases chondrocyte mechano-vulnerability to impact loads

Initial experiments were performed on freshly explanted, *in situ* proximal humeral head articular cartilage to evaluate the baseline mechano-vulnerability of chondrocytes in response to injurious impact loading. There was a statistically significant increase in humeral head chondrocyte cell death following impact loading after culture with Yoda1 relative to controls, indicating that PIEZO1, in part, drives chondrocyte vulnerability to injurious loading (*p* < 0.05, Figures 1B, C). In contrast, there was a statistically significant decrease in humeral head chondrocyte cell death following impact loading after culture with the TRPV4 agonist GSK101, suggesting a chondroprotective role for TRPV4 activation (*p* < 0.01, Figures 1D, E).

### Surgical RCT induces histologic and morphologic changes to the proximal humerus in the early stages of CTA

Rotator cuff surgical ligation and sham surgery (deltoid splitting approach without RC ligation) was performed on 14-week-old mice (Figure 2B). Mice were sacrificed 4-and 14-week post-operatively. At both time points, there was a qualitative histological difference with flattening of the superior portion of the humeral head relative to uninjured contralateral controls and sham surgery (Supplementary Figure S1; Figure 2C). There were notable differences in the gross appearance of the humeral heads from mice that had undergone rotator cuff ligation relative to contralateral controls 14 weeks after injury, such that there was an area of denuded and dehydrated cartilage with underlying bone bruising at the posterior superior quadrant of the humeral head (Figure 2D).

### Surgical RCT increases chondrocyte mechano-vulnerability

**FIGURE 3:**
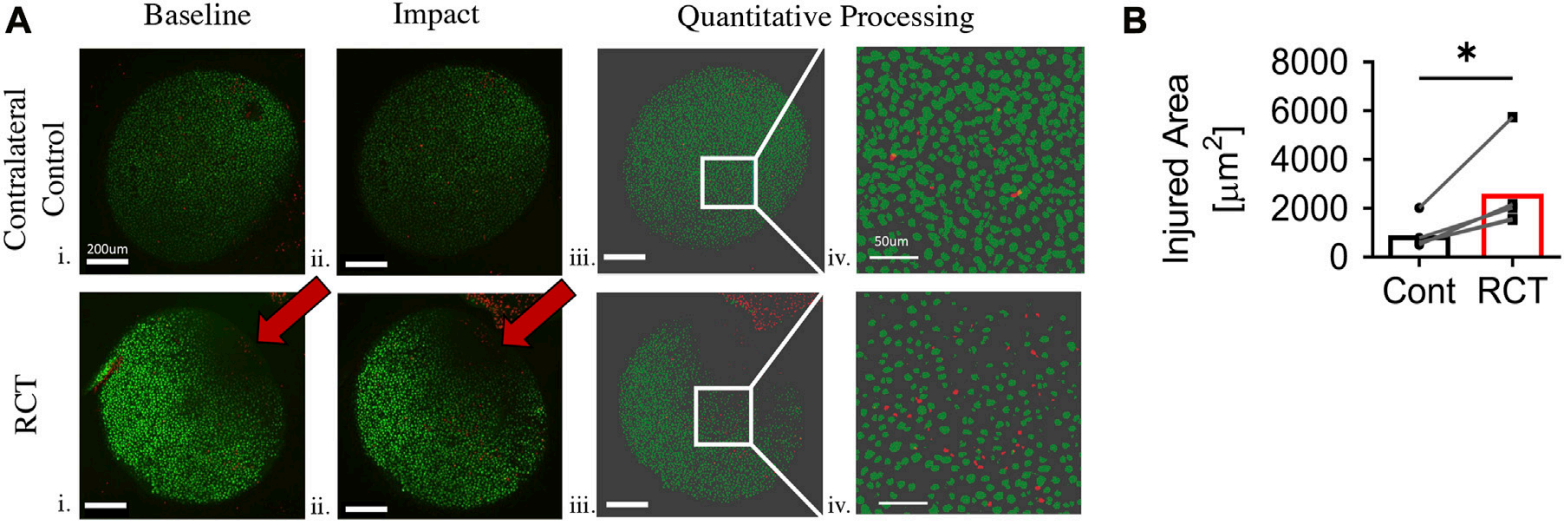
Chondrocyte mechano-vulnerability of humeral heads 14 weeks status post RCT*.* **(A)** Representative images of live/dead assay after 2 mJ impact of humeral cartilage of RCT Control, contralateral limb and RCT. i. Micrograph of before impact. Red arrow indicates bone bruise from RCT, scale bar = 200 μm ii. Micrograph of after impact. Red arrow indicates bone bruise from RCT, scale bar = 200 μm iii. Quantitative processed image from MATLAB analysis of after impact, scale bar = 200 μm iv. Quantitative processed image from MATLAB analysis with increased magnification of injured region, scale bar = 50 μm. **(B)** Quantification of area of cell death for humeral head cartilage subjected to 2 mJ load, *n* = 5 per group, significance of *p < 0.05 with paired Student’s t-test. Cells outside of the sphericity of the humeral head are within the stump of the rotator cuff tendons and joint capsule, which was reflected during dissection.

To assess mechano-vulnerability of humeral chondrocytes, humeri harvested at 4-week (*n* = 3) and 14-week (*n* = 5) status post RCT were subjected to 2mJ of impact loading. There was a significant increase in impact-induced chondrocyte death in proximal humeri of the RCT group *versus* contralateral uninjured controls at both 4- and 14-week post injury (*p* < 0.05, Figure 3 and Supplementary Figure S2). We also observed that chondrocytes were more sparse and smaller in the posterior superior quadrant, corresponding to the area of qualitative changes noted from gross humeral head specimens.

### PIEZO1 expression and activity is decreased in glenohumeral chondrocytes status post RCT

**FIGURE 4:**
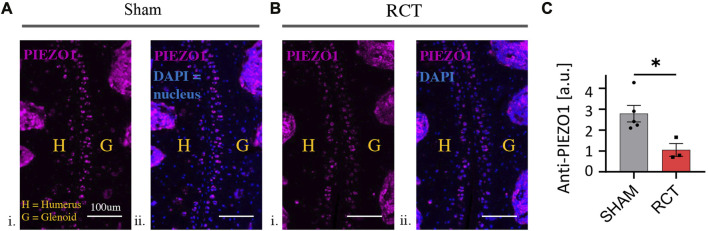
PIEZO1 expression levels 14 weeks status post RCT*.* **(A)** Representative immunofluorescence images of sham control limbs. i. PIEZO1 expression with humerus and glenoid labeled, scale bar = 100 μm ii. PIEZO1 expression with DAPI staining for cell nuclei, with humerus and glenoid labeled, scale bar = 100 μm. **(B)** Representative immunofluorescence images of RCT-injured limbs. i. PIEZO1 expression with humerus and glenoid labeled, scale bar = 100 μm ii. PIEZO1 expression with DAPI staining for cell nuclei, with humerus and glenoid labeled, scale bar = 100 μm. **(C)** Quantification of PIEZO1 expression between control and RCT. Significance of *p < 0.05 with unpaired Student’s t-test, indicating a decrease in expression.

**FIGURE 5:**
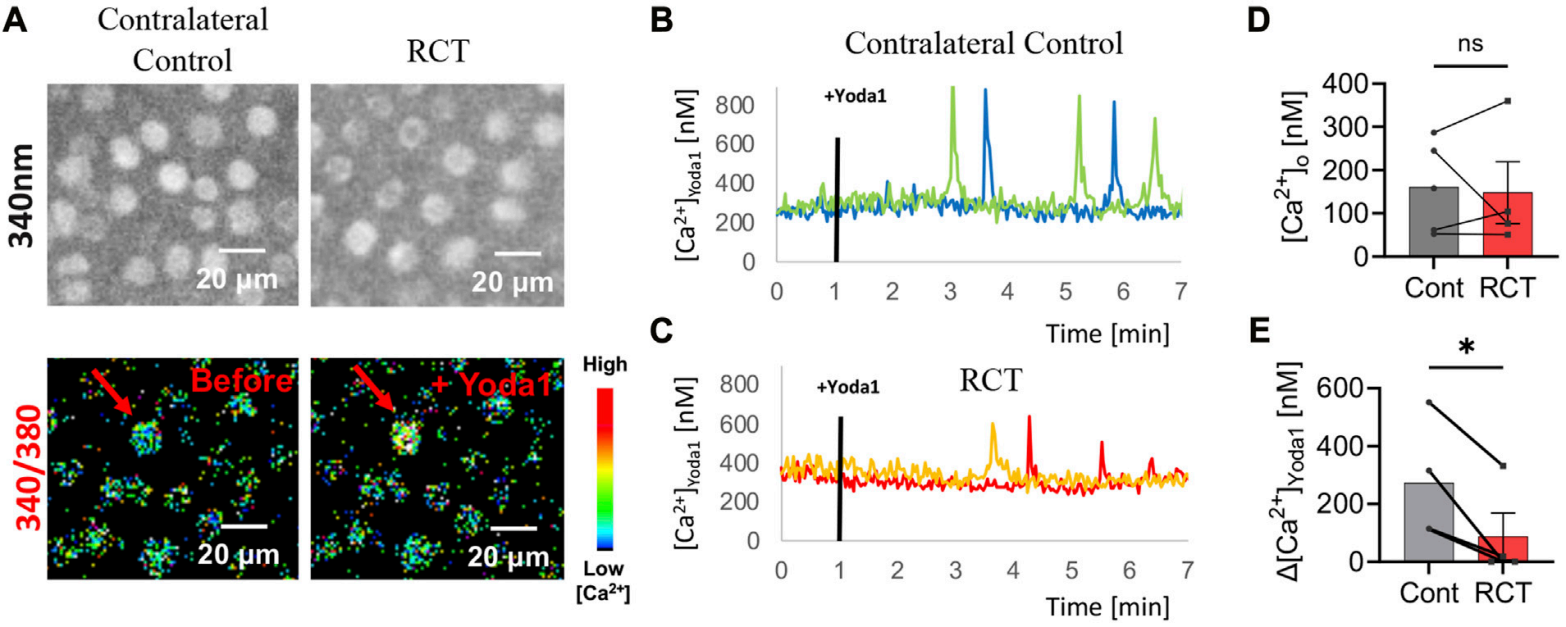
PIEZO1 functional expression 14 weeks status post RCT. **(A)** Representative Fura2 load micrographs: (top) 340 nm excitation, (bottom) ratiometric (340 nm/380 nm) image with pseudo colors. **(B)** Representative intracellular Ca^2+^ concentration of contralateral control mice following Yoda1 treatment 1min *in situ* with blue and green lines representing two of four biologic replicates. **(C)** Representative intracellular Ca^2+^ concentration of RCT injured mice following Yoda1 treatment 1min *in situ* with red and orange lines representing two of four biologic replicates. **(D)** Average resting [Ca^2+^]o and **(E)** average Yoda1 (40 μM)-induced Δ[Ca^2+^]Yoda1 in contralateral control limb compared to RCT-injured limb, no significant differences in [Ca^2+^]o and significantly reduced Δ[Ca^2+^]Yoda1 in RCT-injured limb *versus* contralateral control limb. *p < 0.05, **p < 0.01; paired Student’s t-test.

There was a significant decrease in chondrocyte PIEZO1 protein expression, measured by quantitative immunofluorescence, for glenohumeral joints that underwent RC ligation relative to the sham surgery group (*p* < 0.05, Figure 4). The resting cytosolic calcium concentrations were similar between RCT and contralateral control humeral head chondrocytes, but the Yoda1-induced intracellular calcium flux values were significantly lower in RCT chondrocytes compared to contralateral control limbs (Figure 5). Collectively, these data indicate the reduced functional expression of PIEZO1 channels in humeral chondrocytes status post RCT.

### Chondrocyte PIEZO1 expression and mechano-vulnerability increases following non-invasive ACL injury

In contrast to RCT injured mice, PIEZO1 expression significantly increased in mouse distal femoral cartilage 4 weeks following bilateral ACL injury compared with uninjured control animals (*p* < 0.01, Supplementary Figure S3).

## Discussion

Murine humeral head chondrocytes respond to injurious mechanical loading, in part, through the mechanically gated calcium ion channel PIEZO1. Activation of PIEZO1 with Yoda1 increases the vulnerability of chondrocytes to cell death following impact with injurious loads. Conversely, activation of TRPV4 with GSK101 attenuates cell death following injurious impact loading, which infers a chondro-protective effect. These opposite effects of chemical activation between PIEZO1 and TRPV4 suggest the differential roles of these channels in the trauma-induced chondrocyte damage and death. We previously reported augmented PIEZO1 functional expression in chondrocytes under inflammatory and osteoarthritic conditions in porcine and human knee joints, and further demonstrated that arthritic chondrocytes were more vulnerable to mechanical impact (Lee et al., 2014; Lee et al., 2021). We also recently reported that chondrocyte vulnerability is increased in the osteoarthritic mouse knee following a non-invasive bilateral ACL injury (Kotelsky et al., 2022), which is complemented by an increase in PIEZO1 expression in ACL-injured chondrocytes (Supplementary Figure S3). Through the current study, we discovered that a massive rotator cuff tear similarly significantly increases humeral head chondrocyte vulnerability to mechanical impact relative to uninjured contralateral control limbs. In contrast to the correlation between increased PIEZO1 expression and mechano-vulnerability in the knee, we discovered that humeral head chondrocytes significantly downregulate PIEZO1 expression and activity following a massive RCT. While ACL deficiency increases hyper-physiologic axial and rotational loading of the tibiofemoral joint, rotator cuff deficiency decreases axial loading of the glenohumeral joint secondary to the uncoupling of joint with loss of dynamic stabilization of the rotator cuff. This difference in loading may account for differences in chondrocyte PIEZO1 expression and represents an area of future investigation based on this preliminary study. In the current study, a massive rotator cuff injury increased chondrocyte vulnerability to further injurious loading despite downregulation of both PIEZO1 expression and activity. This finding indicates that factors other than PIEZO1 activity influence chondrocyte homeostasis following injury to the glenohumeral joint. Disruption of the rotator cuff results in derangement of joint biomechanics and metabolism. While not studied in this mouse model, we hypothesize that chondrocytes in post-injury state are more susceptible to cell death due to inflammation from the initial injury and the altered biomechanics, which will be the focus of future investigations utilizing the injury model.

Previous work to define the long-term sequelae of a massive RCT in the mouse model reported that the mouse shoulder progresses through histopathological changes toward cuff tear arthropathy similar to that seen in humans (Zingman et al., 2017). They reported an early increase in cartilage thickness at 14 weeks during a proposed inflammatory period, followed by progressive loss of humeral head sphericity and subchondral bone architecture; pitting, fibrillation, and thinning of articular cartilage; and superior migration of the humeral head with associated acromial acetabularization. At both 4- and 14-week post injury, we identified flattening of the posterior superior portion of the humeral head, which corresponded to an area of bone bruising on explanted specimens. While not assessed in a dynamic environment, we believe that this corresponds to an area of acromial abutment, which could be confirmed future experiments using micro-CT. This finding is consistent with a prior study in rats that reported focal defects of superior humeral head cartilage but no difference in overall cartilage thickness 12 weeks after full-thickness RCT compared with uninjured control limbs (Treviño et al., 2019). Treviño et al. further reported a significant increase in capthesin in humeral head articular cartilage 1 week following RCT and a trend toward increased matrix metalloproteinases in humeral head cartilage up to 12 weeks status post RCT relative to uninjured contralateral control limbs, indicating an early inflammatory and sustained catabolic response of cartilage in response to a torn rotator cuff (Treviño et al., 2019).

Rotator cuff pathology in rodent models have previously been shown to share features seen in humans including bursal-sided healing and scar formation following partial rotator cuff tear (Yoshida et al., 2016); progressive tendon retraction, muscle atrophy, and fatty infiltration following complete tendon transection and/or muscle denervation (Liu et al., 2012; Wang et al., 2018); and impaired gait parameters and shoulder function 6 weeks following massive RCT (Perry et al., 2009; Wang et al., 2018). Despite being quadrupedal, rodents have shoulder anatomy and function that most closely resembles humans among alternative candidate species, excluding non-human primates (Longo et al., 2011; Lebaschi et al., 2016; Liu et al., 2020). Compared with humans, the rodent coracoacromial arch is of similar morphology and orientation (Soslowsky et al., 1996; Derwin et al., 2010), and the excursion of supraspinatus tendon is contained below the acromial arch with ambulation, burrowing, overhead reaching, and climbing (Soslowsky et al., 1996). These anatomical features, along with the histopathological progression toward cuff tear arthropathy make the mouse a suitable candidate species to investigate the response of articular cartilage to a massive rotator cuff tear.

This preliminary animal study has several limitations. Investigation was limited to the assessment of one protein mediated pathway, PIEZO1, for cell vulnerability in a single tissue, articular cartilage. Mechanotransduction is known to be regulated by additional mechanically gated channels (including PIEZO2 and TRPV4) and inflammation, both of which are the topics of ongoing investigation in our laboratory. Further, recent studies have shown that PIEZO1 gain of function mutations in mice increases tendon anabolism, elastic energy storage potential, and compliance (Passini et al., 2021; Nakamichi et al., 2022); meanwhile, Piezo1 conditional knockout results in decreased tendon stiffness (Passini et al., 2021). The role of PIEZO1 in rotator cuff tendon homeostasis, pathology, and repair remains unknown, and future studies to investigate the rotator cuff tendons in addition to the glenohumeral articular cartilage would enhance our understanding of PIEZO1 in a massive rotator cuff tear. This study was also limited by the ability to fully understand the degree to which glenohumeral joint biomechanics are altered following a massive rotator cuff tear. The pathology and histology indicate superior migration of the humeral head within the glenoid and abutment against the acromion similar to the human condition. Gait analysis has previously been shown to be altered following a rotator cuff tear in rodents (Perry et al., 2009), but the intra-articular biomechanics remain unknown. Advances such as dynamic or positional micro-CT may help to elucidate these changes but were unavailable for the current study. Finally, we seek to understand the changes to glenohumeral articular cartilage not only following injury but also after therapeutic intervention. A future aim of the current study is to investigate the histology, biochemistry, and mechanotransduction of articular cartilage following rotator cuff repair in the mouse with the previously described supraspinatus tendon repair model (Lebaschi et al., 2018).

Overall, this study provides preliminary evidence that mouse humeral head chondrocytes respond to injurious mechanical loading through mechanically gated calcium ion channels. In contrast to our hypothesis and our findings in the ACL deficient knee, PIEZO1 expression and activity decreases following a massive rotator cuff tear, likely secondary to decreased axial loading and joint uncoupling following the loss of dynamic stabilization imparted by the rotator cuff tendons. Understanding the pathophysiology of both the articular cartilage and the tendons following a massive rotator cuff tear will elucidate opportunities to promote joint homeostasis following rotator cuff repair and restoration of joint biomechanics.

## Data Availability

The raw data supporting the conclusion of this article will be made available by the authors, without undue reservation.
