# Supplementary figures and images for: PIEZO1 is downregulated in glenohumeral chondrocytes in early cuff tear arthropathy following a massive rotator cuff tear in a mouse model
Source: Front Bioeng Biotechnol. 2023 Sep 5;11:1244975. doi: 10.3389/fbioe.2023.1244975 (PMC10508846; doi:10.3389/fbioe.2023.1244975)

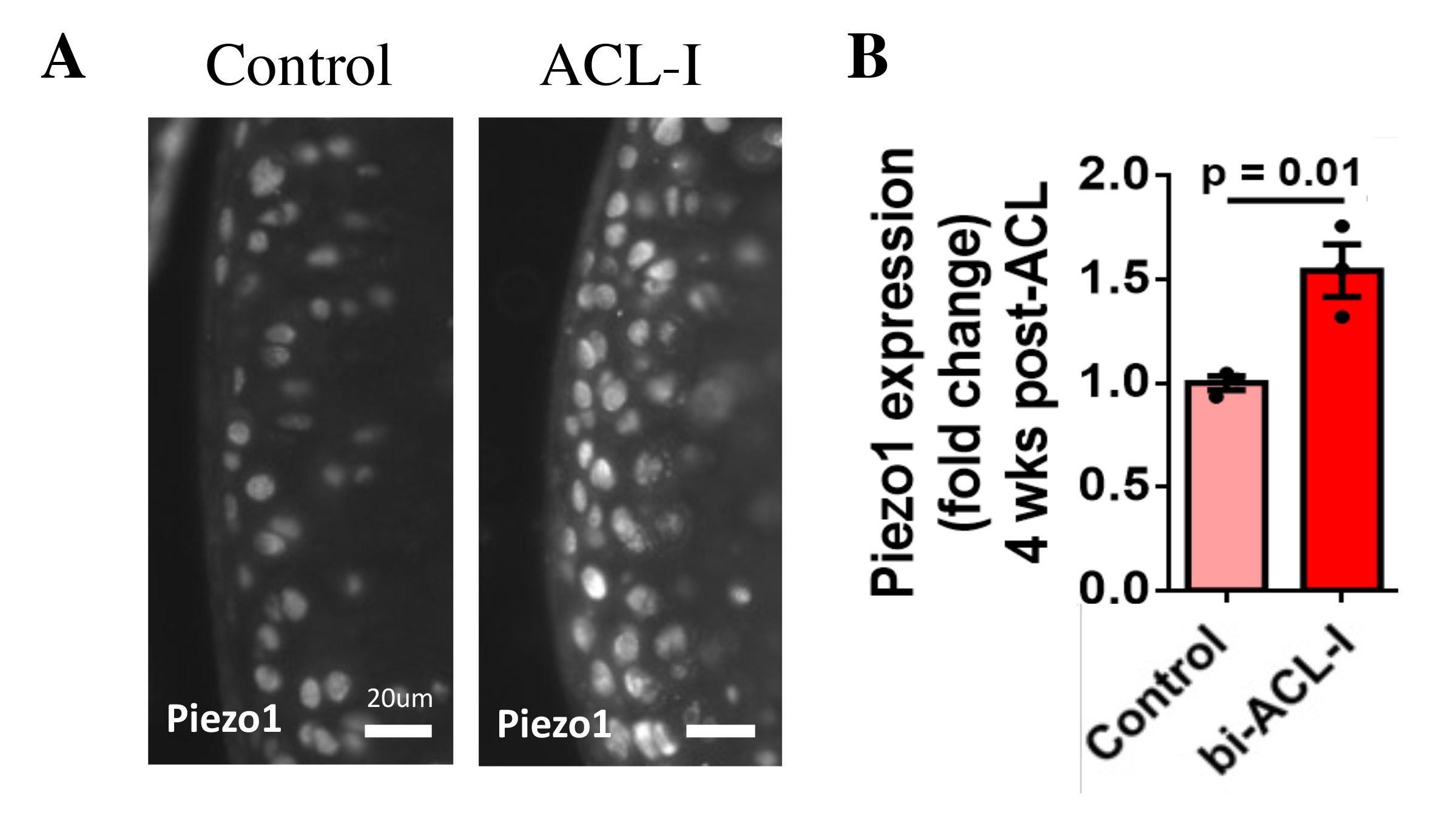

Supplement: Supplementary file 1 [file Image3.jpg]

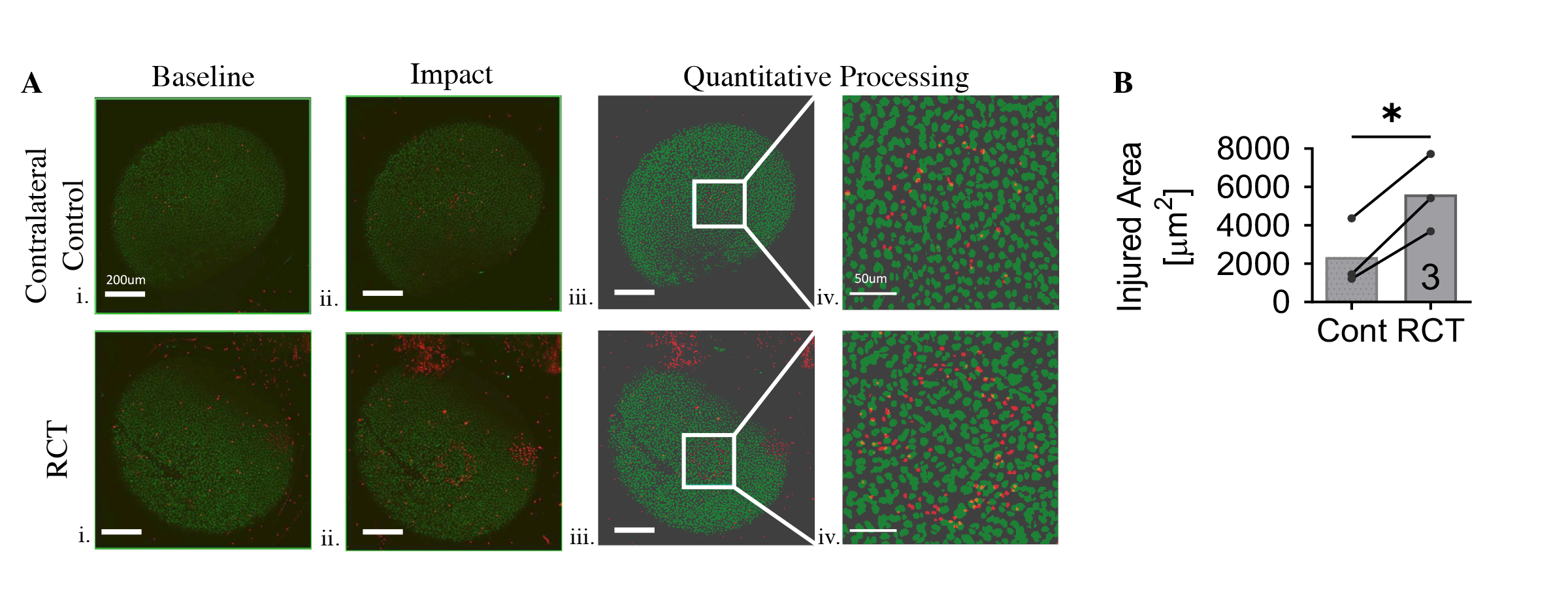

Supplement: Supplementary file 2 [file Image2.jpg]

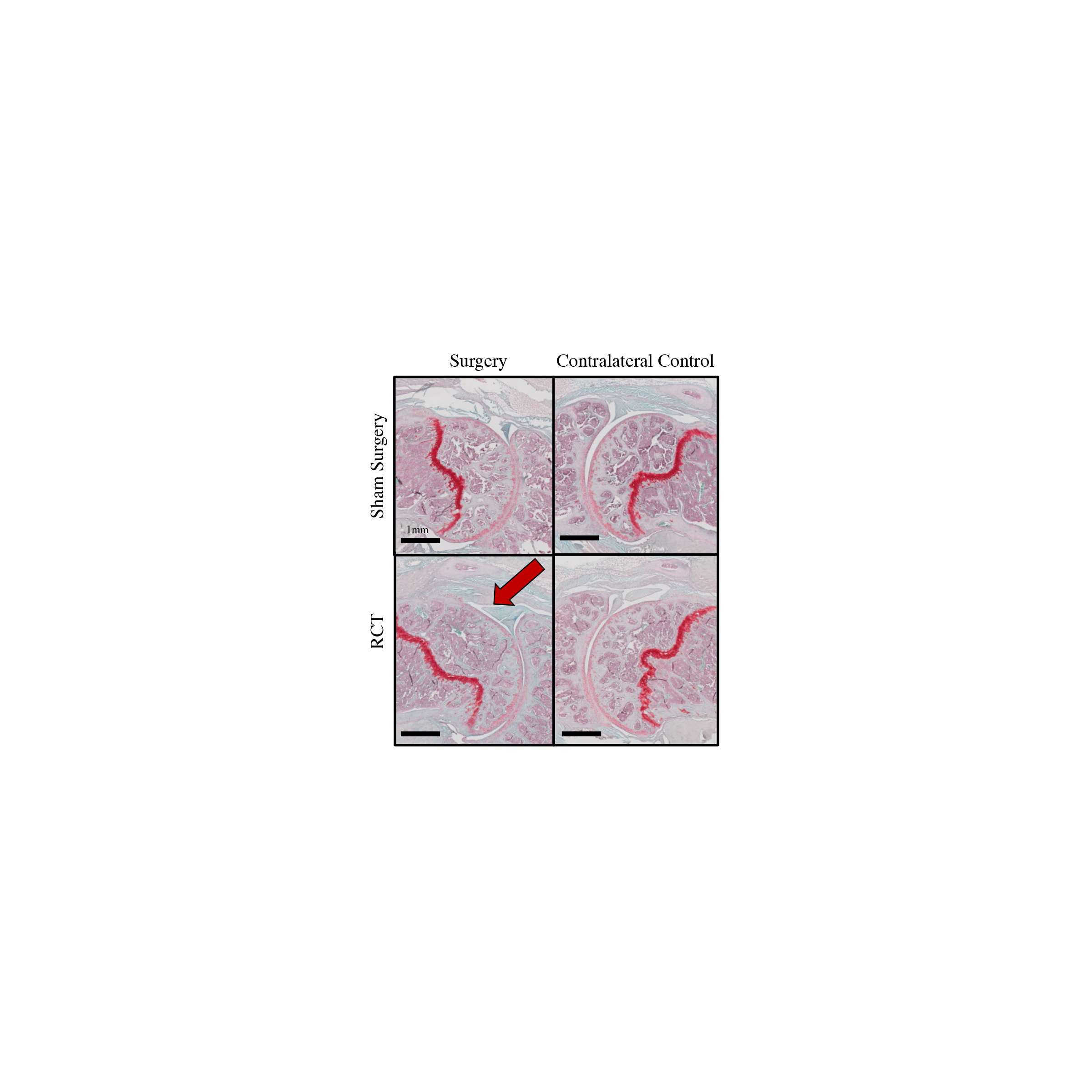

Supplement: Supplementary file 3 [file Image1.jpg]
